# Precautions against Consumption

**Published:** 1890-05

**Authors:** 


### Precautions against Consumption; Circular No. 20, Penna. Board of Health. By Dr. Benjamin Lee, Secretary.

This well-written pamphlet, designed for distribution among the laity, assumes that the cause of consumption is indisputably the bacillus. Upon this it proposes a series of precautions, which are about as follow: Avoid eating meat of cattle known to be tuberculous; avoid living in damp situations or a house having a damp or foul cellar; avoid crowded or ill-ventilated assembly rooms; avoid sleeping in poorly-ventilated apartments; avoid sedentary occupations within-doors.

As drying does not destroy the bacillus, consumptives should avoid spitting where the expectoration may become dry and blown about, and so inhaled. The expectoration should be deposited in a paper cup, that may be bought at drug stores, and burned. When away from home the patient should carry with him a flask containing a five per cent. solution of Carbolic acid or Corrosive Sublimate with which to destroy the infection of his expectoration.

No spittoon should be emptied upon the surface of the ground where domestic animals can get at it. The ordinary spittoon filled with sand or sawdust and found in public houses should be abolished. A consumptive mother should not nurse her own child. The feather duster should not be used about the room occupied by the consumptive. The floor, wood-work, and furniture should be cleaned by wiping with a damp cloth. The patient's clothing should be kept by itself and boiled when washed. Ventilation should not be neglected.

After death the wood-work, furniture, etc., of the room should be disinfected with a solution of Carbolic acid or Corrosive Sublimate. The bed clothing should be disinfected with super-heated steam.

At public houses only china or metallic spittoons should be used, charged with some disinfectant.

The author concludes:

"Should the recommendations of this circular be generally followed, the expectation is not Utopian that the present generation may witness a very considerable reduction in the ravages of what has been not unappropriately termed 'The Great White Plague.'" W. M. J.

